# Risk Factors Associated with Carbapenemase-Producing *Enterobacterales* (CPE) Positivity in the Hospital Wastewater Environment

**DOI:** 10.1128/AEM.01715-20

**Published:** 2020-11-24

**Authors:** Stacy C. Park, Hardik Parikh, Kasi Vegesana, Nicole Stoesser, Katie E. Barry, Shireen M. Kotay, Sarah Dudley, Timothy E. A. Peto, Derrick W. Crook, A. Sarah Walker, Amy J. Mathers

**Affiliations:** a Division of Infectious Diseases and International Health, Department of Medicine, University of Virginia Health System, Charlottesville, Virginia, USA; b Nuffield Department of Medicine, University of Oxford, Oxford, United Kingdom; c National Institute for Health Research Health Protection Research Unit in Healthcare Associated Infection and Antimicrobial Resistance, University of Oxford, Oxford, United Kingdom; d School of Medicine, University of Virginia, Charlottesville, Virginia, USA; e Clinical Microbiology Laboratory, Department of Pathology, University of Virginia Health System, Charlottesville, Virginia, USA; INRS—Institut Armand-Frappier

**Keywords:** carbapenem-resistant *Enterobacterales*, carbapenemase-producing *Enterobacterales*, drain, environmental reservoir, nosocomial infections, sink, sink trap, toilet

## Abstract

Hospital wastewater is an increasingly recognized reservoir for resistant Gram-negative organisms. Factors involved in establishment and persistence of Klebsiella pneumoniae carbapenemase-producing organisms (KPCOs) in hospital wastewater plumbing are unclear. This study was conducted at a hospital with endemic KPCOs linked to wastewater reservoirs and robust patient perirectal screening for silent KPCO carriage. Over 5 months, both rooms occupied and rooms not occupied by KPCO-positive patients were sampled at three wastewater sites within each room (sink drain, sink P-trap, and toilet or hopper). Risk factors for KPCO positivity were assessed using logistic regression. Whole-genome sequencing (WGS) identified environmental seeding by KPCO-positive patients. A total of 219/475 (46%) room sampling events were KPCO positive in at least one wastewater site. KPCO-positive patient exposure was associated with increased risk of environmental positivity for the room and toilet/hopper. Previous positivity and intensive care unit room type were consistently associated with increased risk. Tube feeds were associated with increased risk for the drain, while exposure to patients with Clostridioides difficile was associated with decreased risk. Urinary catheter exposure was associated with increased risk of P-trap positivity. P-trap heaters reduced risk of P-trap and sink drain positivity. WGS identified genomically linked environmental seeding in 6 of 99 room occupations by 40 KPCO-positive patients. In conclusion, KPCO-positive patients seed the environment in at least 6% of opportunities; once positive for KPCOs, wastewater sites are at greater risk of being positive subsequently. Increased nutrient exposure, e.g., due to tube food disposal down sinks, may increase risk; frequent flushing may be protective.

**IMPORTANCE** Klebsiella pneumoniae carbapenemase-producing organisms (KPCOs) are bacteria that are resistant to most antibiotics and thus are challenging to treat when they cause infections in patients. These organisms can be acquired by patients who are hospitalized for other reasons, complicating their hospital stay and even leading to death. Hospital wastewater sites, such as sink drains and toilets, have played a role in many reported outbreaks over the past decade. The significance of our research is in identifying risk factors for environmental positivity for KPCOs, which will facilitate further work to prevent transmission of these organisms to patients from the hospital environment.

## Abstract (precis)

Klebsiella pneumoniae carbapenemase-producing organisms (KPCOs) are bacteria that are resistant to most antibiotics and thus are challenging to treat when they cause infections in patients. These organisms can be acquired by patients who are hospitalized for other reasons, complicating their hospital stay and even leading to death. Hospital wastewater sites, such as sink drains and toilets, have played a role in many reported outbreaks over the past decade. The significance of our research is in identifying risk factors for environmental positivity for KPCOs, which will facilitate further work to prevent transmission of these organisms to patients from the hospital environment.

## INTRODUCTION

Hospital water sources have long been associated with outbreaks due to various pathogens (1). However, in recent years, hospital wastewater in particular has gained recognition as a reservoir and source for nosocomial infections, including multidrug-resistant Gram-negative bacteria (2). Many studies have explored risk factors for patient acquisition of carbapenemase-producing *Enterobacterales* (CPE) (3–5), but risk factors for CPE establishment in hospital wastewater plumbing are less well defined. Designs that promote or disturb drain biofilm, misuse of sinks, and placement of patient care materials adjacent to sinks have all been associated with sink-related infections (6). Factors that facilitate biofilm formation, such as nutrient exposure (7), also plausibly increase risk of CPE establishment and persistence in the wastewater environment. Low frequency of water use and longer columns of stagnant water have also been associated with higher bacterial CFU counts in tap water (8).

Exposure to colonized patients could be another important factor in CPE establishment in hospital wastewater plumbing, but current evidence supporting this is largely anecdotal. Use of sinks to dispose of patient secretions has been associated with sink colonization (9), and environmental surface contamination from CPE-colonized patients appears to be frequent, particularly among “super spreaders” (10). Selective pressure from antibiotic excretion in the urine and feces has been proposed as a potential contributor to the success of multidrug-resistant organisms in hospital plumbing. While studies have demonstrated higher levels of antibiotic residues and relative abundance of antimicrobial resistance genes in hospital wastewater (11–13), studies investigating associations between antibiotic concentrations and specific resistance phenotypes have produced mixed results (14–17).

Persistent low-level transmission of Klebsiella pneumoniae carbapenemase-producing organisms (KPCOs) occurred in our institution for several years and was ultimately linked to wastewater reservoirs (18). Detection of a wastewater source was achieved through a robust perirectal KPCO patient screening program and early adoption of the Centers for Disease Control and Prevention’s (CDC) toolkit to prevent transmission (19), as well as the establishment of environmental sampling protocols and a database to track results. We used these resources together with clinical and patient movement data to investigate the effects of KPCO-positive patients and other clinical factors on KPCO positivity in the wastewater environment. In particular, we used whole-genome sequencing (WGS) to estimate the frequency with which KPCO-positive patients seeded the wastewater environment, and we investigated the impact of exposure to KPCO-positive patients, factors that increase KPCO shedding (e.g., antimicrobial exposure), and patient and staff behaviors that influence interactions with the plumbing on environmental KPCO positivity.

## RESULTS

### Microbiology.

**FIG 1:**
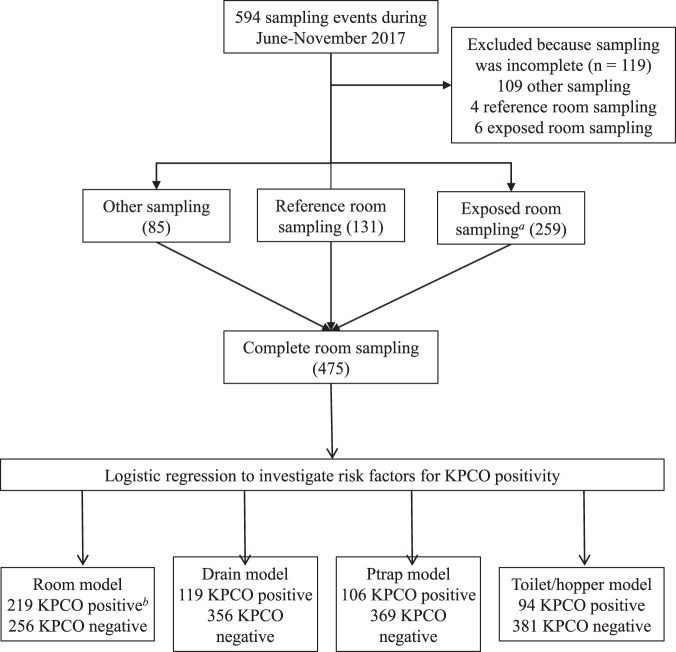
Sampling and environmental KPCO positivity. *a*, 40 KPCO patients occupied 72 unique rooms during the study period. *b*, sampling events wherein at least one wastewater site was positive for KPCO.

A total of 475 complete sampling events (times when samples from the drain, P-trap, and hopper/toilet were all collected from a room) occurred during the study period, of which 219 (46%) were positive for KPCOs from at least one site (Table 1), many with multiple species of KPCOs. A total of 119 (25%) drain samples were positive for KPCOs, as were 106 (22%) P-trap samples and 94 (20%) toilet/hopper samples (Fig. 1). From these 319 KPCO-positive sites, 625 environmental KPCOs were isolated (235 drain, 200 P-trap, and 190 toilet/hopper), 456 (73%) of which were sequenced (201 drain, 133 P-trap, and 122 toilet/hopper) (see Table S5 in the supplemental material). Klebsiella pneumoniae was the most common species among sequenced drain and P-trap isolates (*n* = 59 [29%] and *n* = 40 [30%], respectively). Citrobacter freundii was the most common species among sequenced toilet/hopper isolates (*n* = 33 [27%]). Forty-seven patient-derived KPCO isolates were sequenced; the most common species was K. pneumoniae (*n* = 14 [30%]). Environmental isolates that screened positive for carbapenemase production based on modified carbapenemase inactivation method but that were negative for *bla*_KPC_ based on PCR were further screened for *bla*_NDM_, *bla*_IMP_, *bla*_VIM_, and *bla*_OXA-48_ using PCR; no organisms producing carbapenemases other than KPC were identified.

**TABLE 1:** Characteristics of complete room sampling events

Parameter^*a*^	KPCO-positive sampling events,^*b*^ no. (%) (*n* = 219)	KPCO-negative sampling events, no. (%) (*n* = 256)	Total, no. (%), (*n* = 475)
Any KPCO patient exposure	118 (54)	129 (50)	247 (52)
Any C. difficile patient exposure	20 (9)	29 (11)	49 (10)
Room previously positive	150 (68)	55 (21)	205 (43)
Any antibiotic days	168 (77)	188 (73)	356 (75)
Heater^*c*^	26 (12)	12 (5)	38 (8)
Any tube feed exposure	100 (46)	65 (25)	165 (35)
Any urinary catheter exposure	117 (53)	60 (23)	177 (37)
Any complex wound care	48 (22)	49 (19)	97 (20)
Room type (non-ICU)	125 (57)	223 (87)	348 (73)

a All exposures in the 7 days prior to sampling, except room previously positive, which relates to the last prior sampling of the room. See Table S3 for univariate and multivariate comparisons.

b Sampling events that were positive for at least one wastewater site.

c All rooms on SICU.

**FIG 2:**
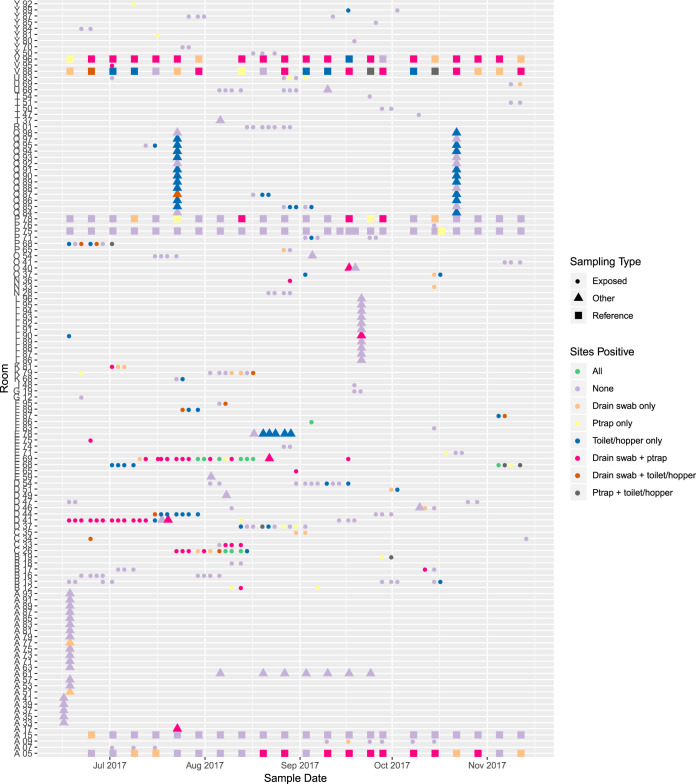
Positivity patterns of complete sampling events. Rooms are arranged geographically on the *y* axis and identified by anonymized alpha-numeric codes. Rooms of the same unit have codes that begin with the same letter. Reference rooms, exposed rooms, and other sampling are indicated by shape. For reference rooms, V96 and V88 were previously consistently positive, P78 and A05 were previously intermittently positive, and P75 and A15 were previously consistently negative.

Patterns of environmental positivity varied markedly (Fig. 2 and Table 2). In previously consistently positive, consistently negative, and intermittently positive reference rooms, 3/42 (7%), 44/46 (96%), and 23/43 (53%) sampling events were negative at all three sites, respectively (Fisher exact test, *P* < 0.001) (Table 2).

**TABLE 2:** Number of wastewater sites positive by sampling type

Parameter	All sites negative, no. (%)	One site positive, no. (%)	Two sites positive, no. (%)	All sites positive, no. (%)
Reference, consistently positive (*n* = 42)	3 (7)	15 (36)	24 (57)	0 (0)
Reference, consistently negative (*n* = 46)	44 (96)	2 (4)	0 (0)	0 (0)
Reference, intermittently positive (*n* = 43)	23 (53)	8 (19)	12 (28)	0 (0)
Exposed rooms (*n* = 259)	133 (51)	65 (25)	50 (19)	11 (4)
Other sampling (*n* = 85)	53 (62)	26 (31)	6 (7)	0 (0)

### Risk factors for environmental KPCO positivity.

**FIG 3:**
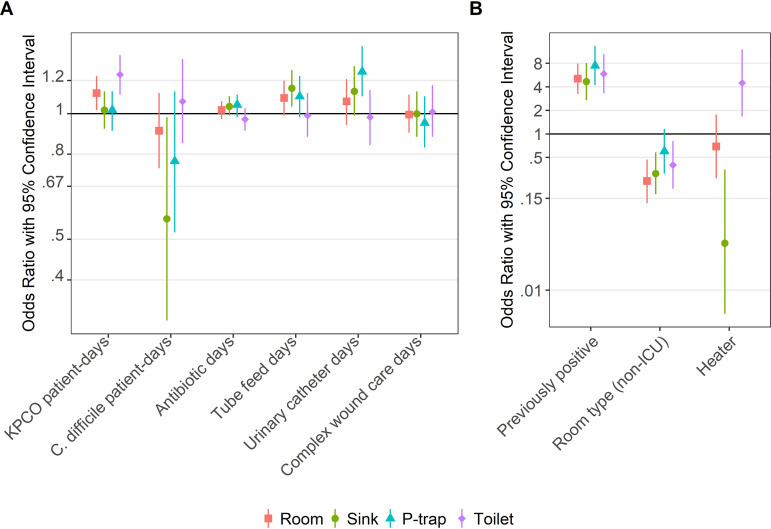
Predictors of KPCO positivity based on multivariate models. Predictor variables were separated into panels A and B based on strength of associations to allow for optimal *y* axis values for visualization. The room model represents the OR for positivity of at least one wastewater site. Observations with heaters (*n* = 38; all rooms in SICU) were not included in the P-trap model due to perfect prediction of heaters as a variable (all observations with heaters were negative for KPCO at the P-trap level).

In multivariate models, some variables had consistent effects on KPCO positivity across wastewater sites, while others had more modest or inconsistent effects (Fig. 3). KPCO patient-days were associated with increased toilet/hopper (odds ratio [OR] = 1.24 and 95% confidence interval [CI] = 1.11 to 1.38) and room level positivity (OR = 1.12 and 95% CI = 1.02 to 1.23), but there was no evidence of association with drain or P-trap positivity (*P* > 0.7 [Tables S1 to S4]). Tube feed days and urinary catheter days modestly increased drain (OR = 1.15 and 95% CI = 1.04 to 1.27 and OR = 1.13 and 95% CI = 0.99 to 1.30, respectively) and P-trap (OR = 1.10 and 95% CI = 0.98 to 1.23 and OR = 1.26 and 95% CI = 1.10 to 1.45) positivity, but there was no evidence of association with toilet/hopper positivity (*P* > 0.7), leading to attenuation of effects of these factors on positivity overall at the room level. There was no evidence of association between positivity and antibiotic days or complex wound care days across all models (*P* > 0.15). C. difficile patient-days were associated with decreased risk of drain positivity (OR = 0.56 and 95% CI = 0.32 to 0.98) and, to a lesser degree, the P-trap (OR = 0.77 and 95% CI = 0.52 to 1.13) but not the toilet/hopper (*P* = 0.57). Positivity at last sampling was consistently associated with substantially increased risks of KPCO positivity in all sites, while non-intensive care unit (non-ICU) room type was consistently associated with decreased risk (Fig. 3B). Heater presence perfectly predicted P-trap negativity for KPCOs; thus, observations with a heater were not included in this model. While heater presence decreased the risk of sink positivity (OR = 0.04 and 95% CI = 0.005 to 0.35), it was associated with significantly increased risk of toilet positivity (OR = 4.48 and 95% CI = 1.67 to 12.03).

### Environmental seeding based on WGS.

**FIG 4:**
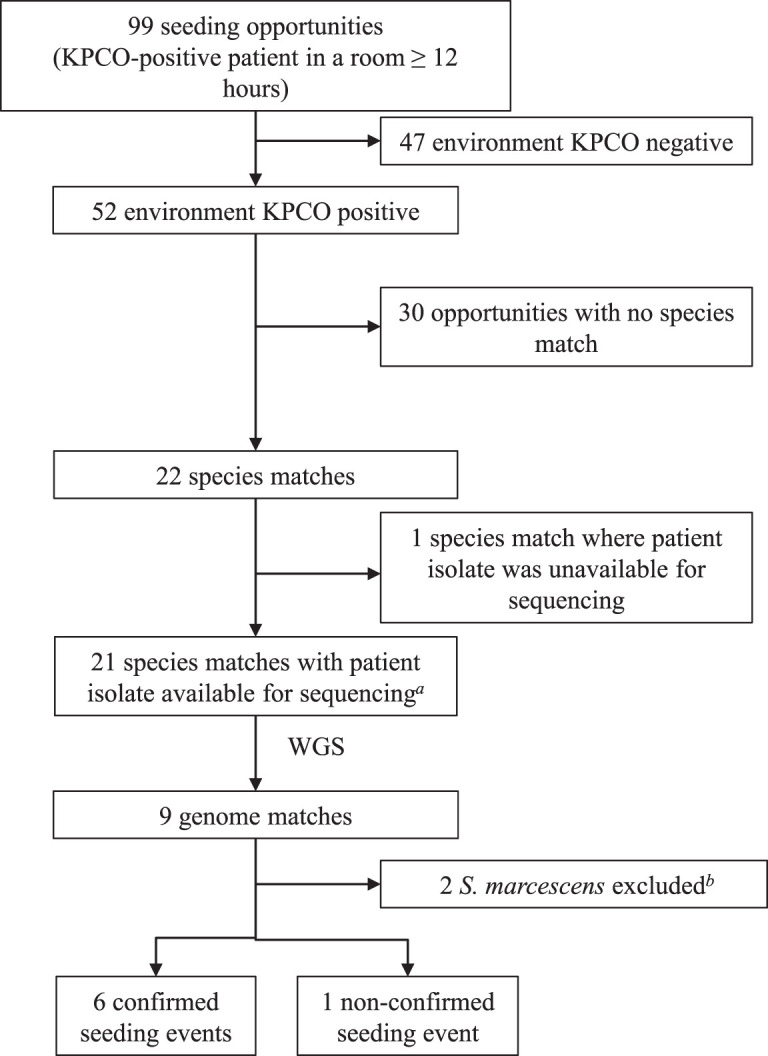
Summary of environmental seeding analysis. *a*, Includes one seeding event in which there were two species matches (patient colonized with multiple KPCOs; two detected in the environment) but only one patient isolate was available for sequencing. *b*, all S. marcescens isolates at the institution were highly related (<20 SNVs across >300 isolates).

During the study period, 40 KPCO-colonized patients occupied 72 distinct rooms for at least 12 h, resulting in 99 seeding opportunities (Fig. 4). A KPCO was detected in the wastewater environment in 52 (53%) seeding opportunities. There was a species match between the patient KPCO and the environmental isolate in 22 (22%) seeding opportunities. One opportunity could not be evaluated genetically, as the patient isolate was unavailable for sequencing. Of the 21 evaluable species matches, 9 were genetic matches. However, two of these were Serratia marcescens and hence discounted because all S. marcescens isolates in our institution are highly genetically related (<20 single nucleotide variations [SNVs] across >300 sequenced isolates). One was also discounted because a matching sequenced historic environmental isolate predated the sequenced patient isolate, resulting in six genomically confirmed seeding events (6 [6%] of 99 seeding opportunities) (Fig. 4). For 2/22 species matches, however, not all isolates corresponding to the KPCO-colonized patient were available for sequencing and may have represented additional genome matches (i.e., giving a total of up to eight genomically confirmed seeding events [8% of seeding opportunities]).

**FIG 5:**
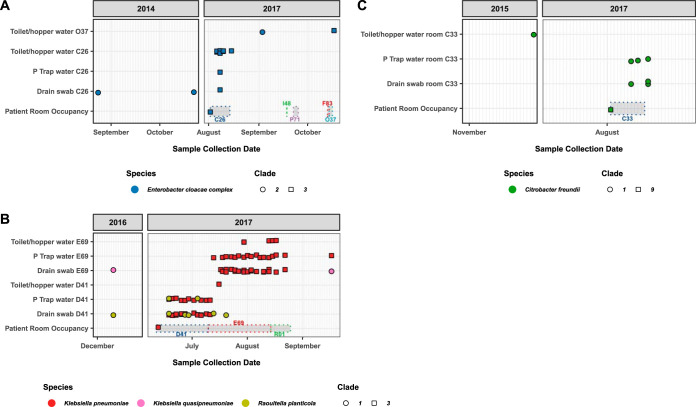
Examples of seeding opportunities. Within each panel the right graph shows isolates from the study period and the left graph shows historical environmental isolates included in the analysis. Isolates are colored by species (or complex in the case of Enterobacter cloacae complex), with shape type delineating genetically related isolates by WGS (clades). Shaded boxes with dotted outlines delineate timing of patient occupations of the exposed rooms. (A) A confirmed seeding event with two rooms seeded several months apart. (B) A confirmed seeding event with two rooms seeded sequentially during the same admission. Clades present historically in the environment were also detected during the study period. (C) A species match that was not a confirmed seeding event based on WGS; the environmental KPCOs detected during the study period were genetically linked to preexisting historical environmental isolates.

Examples of types of genetically confirmed/unconfirmed seeding opportunities are depicted in Fig. 5. Confirmed seeding events include scenarios in which patients seeded different rooms with genetically related KPCOs during different admissions separated by months (Fig. 5A) or during the same admission (Fig. 5B). Of note, environmental sites in rooms were also often positive for other genetically related KPCO over months to years of sampling (Fig. 5A and B). Species matches that were not confirmed as seeding events based on WGS are represented by the scenario depicted in Fig. 5C, in which sequenced environmental C. freundii isolates were genetically related to each other but distinct from the sequenced patient C. freundii isolate.

## DISCUSSION

In this study, we found that exposure to KPCO-positive patients was associated with environmental KPCO positivity for a patient room overall, but only because of an effect on toilet/hopper positivity, with no evidence of effect on drain or P-trap positivity (Fig. 3A). This is plausible, as toilets (and hoppers, which are toilet-like waste disposal units) are the elements most frequently exposed to patient fecal matter, where KPCO patient carriage is most prevalent. However, we did find examples of seeding of all tested wastewater sites from KPCO-positive patients based on genomic data. We additionally found that KPCO-positive patients seeded at least one element of the wastewater environment in at least 6% of opportunities. This is likely an underestimate of the frequency of KPCO-positive patient isolates becoming established in the wastewater environment, as we restricted our definition of seeding events to clonal identity between patient and environmental isolates. This will miss transmission due to horizontal gene transfer via plasmids and mobilization of *bla*_KPC_ between plasmids via transposition and homologous recombination, which contribute to interspecies and intergenus dissemination of *bla*_KPC_ as previously demonstrated (20). Additionally, within phenotypically identical but genotypically mixed populations, colony picks for WGS may have missed environmental isolates that were genetically linked to patient isolates, which could limit confirmation of seeding events. We also excluded a long-standing S. marcescens clone with frequent carriage in both patients and the environment so as to not overestimate contributions from a previously established environmental clone.

Furthermore, we found that previous positivity of a site was consistently and strongly predictive of KPCO positivity upon subsequent sampling (Fig. 3B). This suggests that once a wastewater site is “seeded” with KPCOs, the organisms often thrive and persist. The difficulty many institutions have experienced in clearing the wastewater environment of resistant organisms supports this observation (6). Room type, with non-ICU rooms being much less likely to be positive, was also consistently associated with environmental KPCOs, being most strongly predictive for the room, drain, and toilet/hopper. We have noted this tendency throughout our experience with environmental sampling at our institution: potential explanations for increased KPCO positivity in ICU rooms include decreased patient mobility (resulting in increased direct nursing care and contact with patient bodily fluids), higher severity of illness contributing to microbiome disruption, and higher intestinal load of resistant organisms, such as KPCOs. Of note, increased exposure to KPCO-positive patients and increased exposure to antibiotics (creating more selective pressure) do not seem to explain this room type effect, since these were both included in the multivariate models.

We found an intriguing protective effect of C. difficile patient-days on KPCO positivity at the level of the sink drain and, to a lesser degree, the P-trap. As there is significant overlap between risk factors for C. difficile and CPE, it seems unlikely that this is due directly to the presence of C. difficile. We hypothesize that it is due to differences in the way sinks are used in the rooms of patients known to be positive for C. difficile. While handwashing made up only 4% of activities in a previous observational study of behaviors around ICU sinks, it was anecdotally noted that use of the sink for hand hygiene increased markedly when a C. difficile patient was admitted to the room (21). Current hospital policy considers alcohol gel to be acceptable for hand hygiene for most patients, but for C. difficile-positive patients, soap-and-water hand hygiene is required. The frequent flushing of the pipes with fresh municipal water during hand hygiene may protect against biofilm formation, which was previously demonstrated to be the route for drain colonization following P-trap colonization (7). Tube feed days were also associated with increased KPCO positivity of the drain and, to a slightly lesser extent, the P-trap, which may be due to increased nutrient availability to support biofilm growth when nutrient-rich substances are disposed of down the sink (7, 22). Urinary catheter days were associated with increased KPCO positivity of the P-trap and, to a slightly lesser extent, the sink drain, and we hypothesize that this may reflect decreased patient mobility (and thus less sink usage).

While selective pressure due to antibiotics is frequently mentioned as a factor contributing to the presence of multidrug-resistant organisms in the hospital wastewater, antibiotic days were not an independent predictor in the multivariate models at any level in our study. We focused on systemic antimicrobials, many of which are excreted relatively intact in urine and thus into the wastewater. However, we may have had limited resolution, since we considered only total days of exposure to antibiotics, and different antibiotics may have various influences on KPCO survival in the environment. Additionally, a previous study demonstrated that antibiotics may accumulate in biofilm and be released over time after flushing of a wastewater siphon (13); thus, our 7-day look-back period may not be optimal for examining the relationship between antibiotic use and resistant organisms in the environment.

The sink trap heater-vibration unit, which has been previously described (6), was associated with increased risk for toilet/hopper positivity and decreased risk for sink drain positivity; notably, no P-traps with heaters harbored KPCOs, meaning that this factor could not be included in P-trap models. This likely reflects the nature of the device, which targeted elimination of KPCOs from the P-trap and hence could plausibly affect the associated sink drain but would not be expected to directly affect the rate of toilet/hopper positivity. Of note, the positive association between heater presence and toilet/hopper positivity likely reflects the high background positivity in the unit in which the devices were deployed; we were not able to adjust for this further since heaters were only deployed in this unit.

Our study has several limitations. Some rooms underwent repeated sampling, which we attempted to address with an analysis using a mixed-effects model with room number as a random effect; however, the large number of rooms (123) and high proportion with one or few sampling events led to issues with convergence. Thus, we used a multivariate model with previous positivity and room type, two characteristics most likely to contribute to similarity between samplings of the same room, as covariates. As noted above, 7 days may not be the optimal time frame for assessing the influence of the factors. Finally, our definition of a KPCO-positive patient (any patient with any history of a KPCO-positive culture) may have led to underestimation of the impact of KPCO-positive patient exposure, as several of the KPCO-positive patients had a remote history of KPCOs. However, this is consistent with the definition used at our institution for infection control purposes.

In conclusion, the factors that affect KPCO positivity in the hospital wastewater environment are complex and vary between specific wastewater sites; this is important for those involved in outbreak investigations to consider. KPCO-positive patients seed the wastewater environment at least 6 to 8% of the time, and sites that become positive for KPCOs are likely to be positive thereafter. Therefore, interventions that interrupt transmission to patients or are able to prevent seeding and establishment in wastewater sites may be more successful. Additionally, use of sinks for hand hygiene may be protective, whereas disposal of nutrient-rich substances down sinks may be detrimental. This work provides the basis for several potential infection control and behavioral interventions which could be deployed to reduce the risk of having detectable KPCOs in wastewater reservoirs.

## MATERIALS AND METHODS

### Setting.

Study-specific sampling occurred between June and November 2017 at the University of Virginia hospital, a 619-bed tertiary care hospital with an associated 44-bed long-term acute care hospital (LTACH). A previously described KPCO prevention program was continued throughout the study period (23), including a robust perirectal screening protocol (see the supplemental material). Microbiological processing of perirectal screening samples was performed as previously described (24), except that the modified carbapenemase inactivation method was used for phenotypic detection of carbapenemase instead of the indirect carbapenemase test (25). All hospital rooms contained either a hopper (with a lid and a connected hose) or a toilet (without a lid but in a bathroom separated from the room by a door) that is used for disposal of patient waste, as well as an in-room sink. An educational campaign discouraging the use of intensive care unit (ICU) sinks for activities other than hand hygiene, such as disposal of liquids or storage of patient care items, occurred prior to this study as part of a wastewater-focused bundled intervention (18).

### Environmental sampling.

ESwabs (COPAN, Murrieta, CA) were inserted into drain holes 2.5 cm below the drain for drain samples, and 50 ml of wastewater was collected from the P-trap and toilet or hopper. Samples were transferred into tryptic soy broth with ertapenem for enrichment culture and analyzed for CPE (with *bla*_KPC_ presence determined using PCR) as previously described (18). Exposed rooms were defined as rooms that were occupied for at least 12 h by a KPCO-positive patient and were sampled three times weekly during the patient’s occupation of the room and once after the patient left the room, within 72 h of departure. Rooms at the LTACH were sampled once weekly due to logistical constraints. A KPCO-positive patient was defined as any patient with a history (no time limit) of a culture (clinical or screening) positive for a KPCO, consistent with the definition used for infection control at our institution. Six “reference” rooms were sampled weekly throughout the study period, regardless of exposure to KPCO-positive patients. Reference rooms were selected based on prestudy environmental sampling data to represent three observed patterns of KPCO positivity (consistently positive, consistently negative, and intermittently positive; two reference rooms for each). Other environmental samples taken during the study period were also included, provided that the sampling was a complete sampling event (including a sample from all plumbing sites in the room: sink drain, P-trap, and toilet or hopper). This included sampling that was done within a previously published intervention study (18), which covered the period of this study and entailed installation of sink trap heaters-vibration units (MoveoSiphon ST24; MoveoMed, Dresden, Germany) in the surgical intensive care unit (SICU) rooms, which were in place throughout this study period.

### Risk factors for environmental KPCO positivity.

We used logistic regression to identify risk factors for KPCO positivity in the environment at the level of the room, sink drain, P-trap, and toilet/hopper (Fig. 1). Room positivity was defined as positivity for KPCOs for at least one site. For each environmental sampling event, data from all patients that inhabited the room in the 7 days before sampling was included. Clinical data and patient location data were obtained from an established health system data warehouse. A total of 5% of observations were validated by chart review. As predictors, we included historical environmental CPE positivity in the room, factors which could alter or increase interaction between patient bodily fluids and the wastewater environment, and known risk factors for patient acquisitions of KPCOs (6, 7, 10–13), specifically, KPCO-positive patient-days (captured in hours/minute), C. difficile patient-days (hours/minute), antibiotic patient-days (days), complex wound care patient-days (days), tube feed patient-days (days), urinary catheter patient-days (days), heater presence, room type (ICU or non-ICU), and KPCO positivity at last sampling. C. difficile positivity was defined by a positive PCR (GeneXpert; Cepheid, Sunnyvale, CA) within 30 days of room occupation. The antibiotics included were cefazolin, cefepime, ampicillin-sulbactam, moxifloxacin, trimethoprim-sulfamethoxazole, meropenem, daptomycin, metronidazole, ceftriaxone, piperacillin-tazobactam, ceftazidime-avibactam, vancomycin, and ciprofloxacin. Complex wound care days were defined as presence of any of the following: use of a wound vacuum dressing, bedside debridement, stage four ulcer, unstageable ulcer without stable eschar, full-thickness burn, or skin graft. Heater presence (in all SICU rooms during the study and no rooms in other locations) perfectly predicted P-trap negativity; therefore, observations with a heater (i.e., SICU rooms) were not included in the P-trap model. No variable selection was performed (i.e., all variables were included in the multivariate model regardless of *P* value in the univariate model). A sensitivity analysis using multivariable fractional polynomials did not identify any nonlinear associations with the continuous predictors, which were therefore retained as linear in all models. Analysis used the stats, arm, car, mfp, and ggplot2 packages in R, version 3.5.1.

### WGS and bioinformatics analysis.

Seeding opportunities, defined as occupation of a room by a KPCO-positive patient for at least 12 h, were further characterized using WGS. All available isolates from KPCO-colonized patients underwent WGS, as did all environmental KPCO isolates from the corresponding exposed rooms (excluding only duplicate isolates of the same species from the same sampling site and date due to funding constraints). DNA was extracted using a commercial kit (QuickGene DNA tissue kit S; Fujifilm, Japan) per the manufacturer’s instructions, with an additional mechanical lysis step (FastPrep; MP Biomedicals, USA) following chemical lysis. Sequencing was performed on an Illumina HiSeq 2000 instrument (Illumina, San Diego, CA) as previously described (20). See the supplemental material for bioinformatics analysis (26–30). Isolates within 25 SNVs were considered to be genomically related except for *Enterobacter* spp., for which isolates within 100 SNVs were considered to be genomically related due to lack of a closely related reference.

### Environmental seeding.

We defined a species match when any species harbored by a KPCO-positive patient matched any species of KPCO (determined by matrix-assisted laser desorption ionization–time of flight mass spectrometry [MALDI-TOF MS]) found in the environment during or after the exposure to the patient. When a species match occurred, historical environmental sampling data were checked for prior isolation of the same species in the room, and WGS were compared if available. Patient and environmental isolates were considered a genome match when genetically related based on WGS (as defined above). For genome matches where the species had been detected in the room historically but no sequenced historical isolate was available, the most recent historical isolate was sequenced for comparison. We defined a confirmed seeding event as a genome match where the environmental isolate was distinct from historical environmental isolates of the same species identified prior to KPCO patient occupancy (Fig. 4).

### Ethics statement.

Data were collected from the infection prevention and control database established under the University of Virginia Institutional Review Board protocol 18393. Data analysis and patient chart review were done under protocols 18776 and 13558.

### Data availability

The whole-genome sequencing data for all isolates in this study are available at NCBI’s Sequence Read Archive (SRA) under the BioProject accession numbers PRJNA411762, PRJNA246471, and PRJNA611540.

## Supplementary Material

Supplemental file 1
