# Supplementary material for: Risk Factors Associated with Carbapenemase-Producing Enterobacterales (CPE) Positivity in the Hospital Wastewater Environment
Source: Appl Environ Microbiol. 2020 Nov 24;86(24):e01715-20. doi: 10.1128/AEM.01715-20 (PMC7688209; doi:10.1128/AEM.01715-20)
Supplement: Supplemental file 1 [file AEM.01715-20-s0001.pdf]

SUPPLEMENTAL APPENDIX

Indications for Perirectal CPE Screening per Protocol

1) on admission for patients from countries with endemic CPE or patients admitted from LTACHs
2) on admission to the medical intensive care unit (MICU) and surgical intensive care unit (SICU)
3) weekly screening in the MICU, SICU, and units where any known KPCO-colonized patient was present.

Bioinformatics Analysis

Raw paired-end Illumina reads for each isolate were quality filtered to remove adapter sequences and trim low-quality tail-ends using Trim Galore. Species identification was performed directly by calculating genomic distances between isolate read sets and a curated bacterial whole-genome reference database prepared from NCBI's RefSeq genomes (accessed: 06/2019), using MASH.(1) To compare sets of isolates within the same species, a common reference strain with the shortest distance to all isolates in the comparison was determined by pairwise genomic distances. Reads were aligned to the reference strain genome using *bwa mem*,(2) followed by single-nucleotide variant (SNV) detection using a Bayesian haplotype-based variant detector.(3) Pairwise SNV comparison was performed at the whole-genome level using multiple-sequence alignment, and a SNV-distance matrix generated for each bacterial species. Isolates within 25 SNVs were considered to be genomically related except for *Enterobacter* spp., where isolates within 100 SNVs were considered to be genomically related due to lack of a closely related reference. A maximum likelihood phylogeny was generated using IQ-TREE,(4) with a general time-reversible substitution model + ascertainment bias correction (GTR+ASC) and ultrafast bootstrapping of 1000 replicates, followed by joint ancestral state reconstruction.(5)

Supplemental Table 1. Predictors of KPCO positivity at the room level

	**N (%) or median (IQR)**		**Univariate p**	**Multivariate model**	
	**KPCO positive sampling events (n=219)**	**KPCO negative sampling events (n=256)**		**Odds ratio (95% CI)**	**p**
**KPCO patient-days**	1·1 (0-6·3)	0·5 (0-3·3)	0·002	1·12 (1·02-1·23)	0·01
***C. difficile* patient-days**	0 (0-0)	0 (0-0)	0·77	0·91 (0·74-1·12)	0·35
**Room previously positive**	150 (68%)	55 (21%)	<0·001	5·09 (3·26-7·95)	<0·001
**Antibiotic days**	4 (1-8)	3 (0-7)	0·001	1·02 (0·97-1·07)	0·45
**Heater**	26 (12%)	12 (5%)	0·005	0·69 (0·27-1·77)	0·44
**Tube feed days**	0 (0-4)	0 (0-1)	<0·001	1·09 (0·99-1·20)	0·07
**Urinary catheter days**	1 (0-3)	0 (0-0)	<0·001	1·07 (0·94-1·21)	0·32
**Complex wound care days**	0 (0-0)	0 (0-0)	0·10	0·995 (0·90-1·11)	0·93
**Room type (non-ICU)**	125 (57%)	223 (87%)	<0·001	0·25 (0·13-0·47)	<0·001

Note: all exposures in the 7 days prior to sampling, except room previously positive which relates to the last prior sampling of the room. For continuous factors, odds ratios are per day longer.

Supplemental Table 2. Predictors of KPCO positivity at the sink drain level

	N (%) or median (IQR)		Univariate p	Multivariate model	
	KPCO positive sampling events (n=119)	KPCO negative sampling events (n=356)		Odds ratio (95% CI)	p
**KPCO patient-days**	0 (0-7)	0·9 (0-3·7)	0·007	1·02 (0·92-1·13)	0·71
***C. difficile* patient-days**	0 (0-0)	0 (0-0)	0·02	0·56 (0·32-0·98)	0·04
**Drain previously positive, n(%)**	66 (56%)	41 (12%)	<0·001	4·67 (2·70-8·07)	<0·001
**Antibiotic days**	4 (0-10)	3 (1-7)	0·007	1·04 (0·99-1·10)	0·15
**Heater, n(%)**	1 (0·8%)	37 (10%)	0·01	0·04 (0·005-0·35)	0·003
**Tube feed days**	1 (0-7)	0 (0-1)	<0·001	1·15 (1·04-1·27)	0·009
**Urinary catheter days**	1 (0-5)	0 (0-1)	<0·001	1·13 (0·99-1·30)	0·06
**Complex wound care days**	0 (0-6)	0 (0-0)	<0·001	0·999 (0·88-1·13)	0·99
**Room type (non-ICU), n(%)**	72 (61%)	276 (78%)	<0·001	0·31 (0·17-0·58)	<0·001

Supplemental Table 3. Predictors of KPCO positivity at the P-trap level

	N (%) or median (IQR)		Univariate p	Multivariate model	
	KPCO positive sampling events (n=106)	KPCO negative sampling events (n=331)		Odds ratio (95% CI)	p
**KPCO patient-days**	1·8 (0-7)	0·9 (0-3·8)	0·002	1·02 (0·91-1·13)	0·74
***C. difficile* patient-days**	0 (0-0)	0 (0-0)	0·07	0·77 (0·52-1·13)	0·18
**P-trap previously positive, n(%)**	63 (59%)	34 (10%)	<0·001	7·49 (4.20-13.37)	<0·001
**Antibiotic days**	4 (0-10)	3 (0-7)	<0·001	1·05 (0·98-1·11)	0·15
**Tube feed days**	1 (0-7)	0 (0-1)	<0·001	1·10 (0·98-1·23)	0·10
**Urinary catheter days**	2 (0-6)	0 (0-0)	<0·001	1·26 (1·10-1·45)	0·001
**Complex wound care days**	0 (0-6·8)	0 (0-0)	<0·001	0·95 (0·83-1·10)	0·52
**Room type (non-ICU), n(%)**	70 (66%)	278 (84%)	<0.001	0·60 (0·31-1·16)	0·13

Observations with heater (n=38) not included in P-trap model due to perfect prediction of heater (all observations with heater were negative for KPCO at the P-trap level).

Supplemental Table 4. Predictors of KPCO positivity at the toilet/hopper level

	N (%) or median (IQR)			Multivariate model	
	KPCO positive sampling events (n=94)	KPCO negative sampling events (n=381)		Odds ratio (95% CI)	p value
**KPCO patient-days**	2·1 (0-5·6)	0 (0-3·8)	0·009	1·24 (1·11-1·38)	<0·001
***C. difficile* patient-days**	0 (0-0)	0 (0-0)	0·003	1·07 (0·85-1·35)	0·57
**Toilet/hopper previously positive, n(%)**	50 (53%)	41 (11%)	<0·001	5·88 (3·31-10·43)	<0·001
**Antibiotic days**	4 (2-8)	3 (0-7)	0·23	0·97 (0·91-1·03)	0·31
**Heater, n(%)**	26 (28%)	12 (3%)	<0·001	4·48 (1·67-12·03)	0·003
**Tube feed days**	0 (0-3)	0 (0-2)	0·45	0·99 (0·88-1·12)	0·92
**Urinary catheter days**	1 (0-3)	0 (0-1)	0·03	0·98 (0·84-1·14)	0·76
**Complex wound care days**	0 (0-0)	0 (0-0)	0·47	1·01 (0·88-1·17)	0·86
**Room type (non-ICU), n(%)**	45 (48%)	303 (80%)	<0·001	0·40 (0·20-0·81)	0·01

Supplemental Table 5. Species identification of sequenced isolates

Isolate Source	KPCO positive	Isolates (sequenced)	Species (based on whole genome sequencing)	N (%)
Drain swab	119/475	235 (201)		201
			Aeromonas hydrophila	4 (2)
			Citrobacter farmeri	5 (3)
			Citrobacter freundii	21 (10)
			Enterobacter asburiae	3 (1)
			Enterobacter cloacae	2 (1)
			Enterobacter soli	1 (0·5)
			Enterobacteriaceae (unspecified species)	14 (7)
			Klebsiella michiganensis	3 (1)
			Klebsiella oxytoca	6 (3)
			Klebsiella pneumoniae	59 (29)
			Klebsiella quasipneumoniae	2 (1)
			Metakosakonia spp.	4 (2)
			Phytobacter ursingii	53 (26)
			Raoultella ornithinolytica	3 (1)
			Raoultella planticola	7 (3)
			Serratia marcescens	14 (7)
P-trap water	106/475	200 (133)		133
			Citrobacter farmeri	3 (2)
			Citrobacter freundii	10 (8)
			Enterobacter asburiae	3 (2)
			Enterobacter cloacae	2 (2)
			Enterobacter hormaechei	1 (1)
			Enterobacter spp.	2 (1)
			Enterobacteriaceae (unspecified species)	18 (14)
			Klebsiella pneumoniae	40 (30)
			Klebsiella quasipneumoniae	2 (2)
			Klebsiella variicola	5 (5)
			Metakosakonia spp.	6 (5)
			Phytobacter ursingii	29 (22)
			Raoultella ornithinolytica	1 (1)
			Raoultella planticola	4 (3)
			Serratia marcescens	7 (5)
Toilet/hopper water	94/475	190 (122)		122
			Aeromonas hydrophila	12 (10)
			Citrobacter freundii	33 (27)
			Citrobacter portucalensis	6 (5)
			Citrobacter spp.	3 (2)
			Enterobacter asburiae	21 (17)

**Isolate Source**	**KPCO positive**	**Isolates (sequenced)**	**Species (based on whole genome sequencing)**	**N (%)**
			Enterobacter cloacae	12 (10)
			Enterobacter hormaechei	2 (2)
			Enterobacter roggenkampii	1 (1)
			Enterobacter soli	2 (2)
			Enterobacteriaceae (unspecified species)	2 (12)
			Klebsiella aerogenes	1 (1)
			Klebsiella michiganensis	1 (1)
			Klebsiella oxytoca	4 (3)
			Klebsiella pneumoniae	8 (7)
			Phytobacter ursingii	4 (3)
			Serratia marcescens	10 (8)
Patient				47
			Citrobacter farmeri	2 (4)
			Citrobacter freundii	7 (15)
			Citrobacter portucalensis	1 (2)
			Citrobacter spp.	1 (2)
			Enterobacter asburiae	2 (4)
			Enterobacter cloacae	2 (4)
			Enterobacter hormaechei	2 (4)
			Escherichia coli	4 (9)
			Klebsiella oxytoca	2 (4)
			Klebsiella pneumoniae	14 (30)
			Klebsiella quasipneumoniae	1 (2)
			Klebsiella variicola	1 (2)
			Phytobacter ursingii	1 (2)
			Raoultella ornithinolytica	1 (2)
			Raoultella planticola	1 (2)
			Serratia marcescens	5 (11)

Note: Number of isolates exceeds number of KPCO positive sampling events, as KPCO positive sites may have more than one KPCO at any given sampling point.

1. Ondov BD, Treangen TJ, Melsted P, Mallonee AB, Bergman NH, Koren S, Phillippy AM. 2016. Mash: fast genome and metagenome distance estimation using MinHash. Genome Biol 17:132.
2. Li H. 2013. Aligning sequence reads, clone sequences and assembly contigs with BWA-MEM.
3. Garrison E, Marth G. 2012. Haplotype-based variant detection from short-read sequencing.
4. Nguyen L-T, Schmidt HA, von Haeseler A, Minh BQ. 2015. IQ-TREE: a fast and effective stochastic algorithm for estimating maximum-likelihood phylogenies. Mol Biol Evol 32:268–274.
5. Pupko T, Pe’er I, Shamir R, Graur D. 2000. A fast algorithm for joint reconstruction of ancestral amino acid sequences. Mol Biol Evol 17:890–896.
